# Syndromic capillary malformation with leg length discrepancy: Parkes-Weber syndrome treated by embolization, chemotherapy and Ilizarov technique

**DOI:** 10.1186/s41065-025-00474-9

**Published:** 2025-06-07

**Authors:** Ren Cai, Yifeng Han, Mao Ye, Xitao Yang, Hao Gu, Xiaojie Yue, Xiong Zhao, Xindong Fan, Dachuan Sun, Jiaxue Zhu

**Affiliations:** 1 Department of Interventional Therapy, Multidisciplinary Team of Vascular Anomalies, Shanghai Ninth People’s Hospital, Shanghai Jiao Tong University School of Medicine, Shanghai, PR China; 2 Department of Orthopedics, Fengcheng Hospital, Shanghai Ninth People’s Hospital, Shanghai Jiao Tong University, Shanghai, PR China; 3 Department of Burn and Plastic Surgery, The Children’s Hospital, Zhejiang University School of Medicine, National Clinical Research Center for Child Health, Hangzhou, PR China

**Keywords:** Capillary Malformation, Leg Length Discrepancy, Klippel-Trenaunay Syndrome, Diffuse Capillary Malformation with Overgrowth, Parkes-weber Syndrome, Ilizarov technique

## Abstract

Capillary malformations (CMs) are congenital low-flow vascular anomalies caused by dilated capillaries. Leg length discrepancy (LLD) is the condition characterized by unequal lower limb lengths, leading to functional and postural challenges. Capillary malformation with leg length discrepancy (CM-LLD) formally reveals syndrome such as Klippel-Trenaunay syndrome and Diffuse Capillary Malformation Overgrowth. In this study, we report a syndromic capillary malformation with leg length discrepancy diagnosed as Parkers-Weber Syndrome by radiology and genetic study. This study emphases on understanding the association between CM-LLD, ensuring timely genetic testing, intervention, optimizing functional outcomes, and improving quality of life for individuals with Parkes-Weber syndrome.

## Introduction

Leg length discrepancy(LLD), or termed “anisomelia”, a condition characterized by unequal lower limb lengths, which arises from congenital anomalies, traumatic injuries, or developmental disorders, leading to functional and postural challenges [1]. Capillary malformations (CMs), are congenital and progressive low-flow vascular anomalies caused by dilated capillaries, affecting in 3–5 out of every 1000 newborns [2]. According to the updated ISSVA classification, CMs with hypertrophy or extracutaneous disease are divided into the section of syndromic port-wine CM, which revealed the Diffuse Capillary Malformation Overgrowth (DCMO) [3]. In the former literature, the linkage of CM-LLD was frequently observed in syndromes such as Klippel-Trenaunay syndrome(KTS), where CMs coincide with venous anomalies and limb overgrowth [4]. Herein, we report a medical case of special syndromic Capillary Malformation with Leg Length Discrepancy (CM-LLD), which was neither DCMO nor KTS, but Parkers-Weber Syndrome (PKWS). And we put forward our aspect of differential diagnoses and multidisciplinary management approach for the outcome of the patient.

## Report

**Fig. 1:**
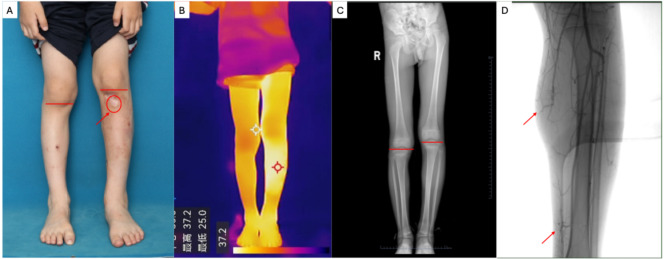
Clinical manifestation and radiograph of the patient. **A:** leg length discrepancy with capillary malformation. The red arrow and circle showed formal ischemia, ulcer and scar lesion. The red line showed the different knee level. **B:** The temperature of capillary malformation is higher than adjacent tissue and the area of the healthy leg in the thermometric indicator. **C:** Digital radiography of the lower extremities showed longer tibia in the lesional leg than the healthy one

A 7-year-old boy was referred to our clinic with a history of congenital erythema on his left lower limb and leg length discrepancy (LLD). He had previously been diagnosed with capillary malformation (CM) and Klippel-Trenaunay syndrome (KTS) at another institution. His vascular lesion became warmer following physical activity or emotional excitement. Over the years, the lesion had darkened progressively and was associated with pain and ulceration, significantly impacting his daily life. On physical examination, the affected left lower leg was 2 °C warmer and 3.5 cm longer than the contralateral limb, leading to mild scoliosis. Clinical photographs and radiographs of the lower extremities were obtained (Fig. 1A–C).

A skin biopsy was performed for histopathological analysis, and targeted next-generation sequencing (NGS) with a depth of 20,000× was conducted. A pathogenic RASA1 loss-of-function mutation (p.Arg398*) was identified in both peripheral blood and tissue samples, without evidence of a second-hit mutation. The patient went through Digital Subtraction Angiograph (DSA) where micro-arteriovenous fistula was detected (Fig. 1D). The final diagnosis was Parkes-Weber Syndrome (PKWS). Initial treatment involved interventional embolization by absolute ethanol every 6 month and targeted chemotherapy of trametinib, the MEK inhibitor of 0.025 mg/qd to address the micro-arteriovenous fistula.

## Discussion

Capillary malformations (CMs) are low-flow vascular anomalies caused by ectatic capillary vessels. They are typically present at birth, tend to enlarge with growth, do not regress spontaneously, and exhibit normal endothelial cell turnover rates [5]. Leg length discrepancy(LLD) refers to a condition in which one leg is shorter than the other, due to either structural differences or functional factors. Even minor discrepancies(5–20 mm) can disrupt biomechanical alignment, potentially leading to chronic pain, osteoarthritis, and postural dysfunction, thereby significantly affecting quality of life. Capillary malformations can present with variable appearances and associated with syndromic conditions. When CMs presents in combination with LLD (CM-LLD), especially at initial clinical evaluation, syndromic CM should be considered in the differential diagnosis.

The differential diagnosis includes Klippel-Trénaunay syndrome(KTS), diffuse capillary malformation with overgrowth(DCMO), and Parkes-Weber syndrome (PKWS). KTS is characterized by vascular stains, lymphatic malformations with or without venous anomalies, and soft tissue and/or bony overgrowth [6]. DCMO is a rare disorder presenting with widespread capillary malformations and overgrowth of soft tissue and/or bone [7]. PKWS involves capillary malformations and micro-arteriovenous fistulas(mAVFs), often with associated lymphatic anomalies, and is typically present from birth [8]. All three conditions may feature CM-LLD. Among them, the association between CM-LLD is particularly notable in KTS [6]. According to the updated ISSVA classification, CMs associated with hypertrophy are classified as syndromic port-wine CMs, such as those seen in DCMO. Although CM-LLD could be observed in all three syndromes, they differ in clinical manifestations, radiologic feature, and genetic study.

**Fig. 2:**
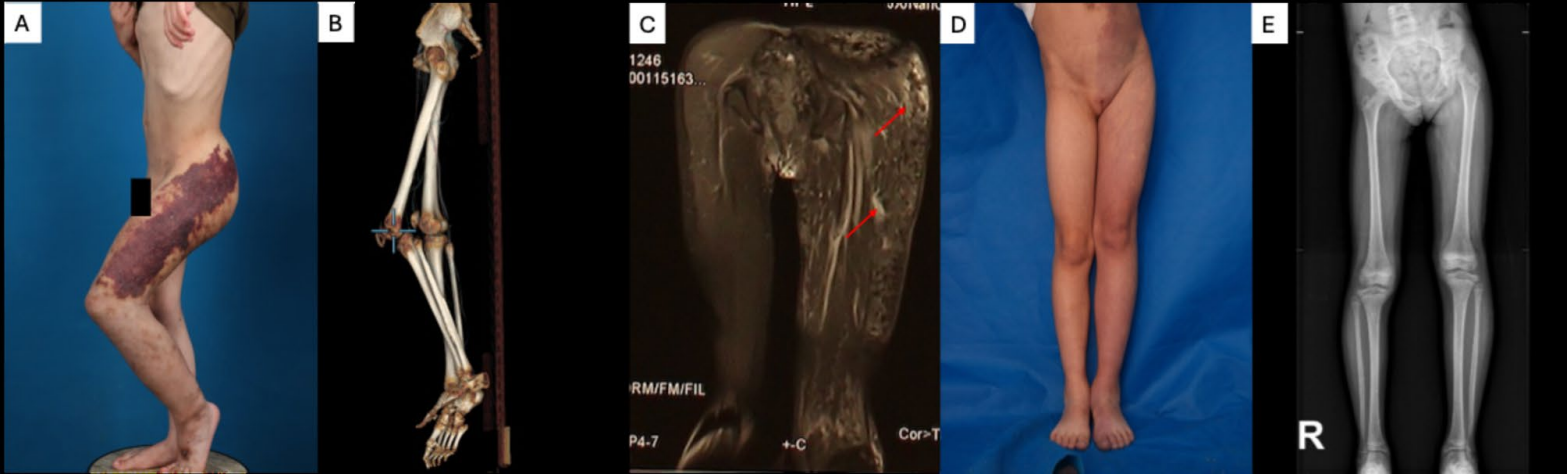
Capillary Malformation with Leg Length Discrepancy(CM-LLD) in Klipple-Trenaunay Syndrome(KTS) and Diffuse Capillary Malformation with Overgrowth(DCMO). **A:** The Clinical manifestation of KTS: lymphatic blister, capillary malformation, and leg length discrepancy. **B:** CT scan showed the bone hypertrophy and leg length discrepancy. **C:** MRI revealed the lymphatic malformation(red arrows show); **D:** The Clinical manifestation of DCMO: an extensive, diffuse, reticulate capillary malformation with bony hypertrophy; **E:** Digital radiography of the lower extremities showed longer tibia in the lesional leg than the healthy one

From a clinical standpoint, in addition to CM-LLD, each syndrome has a predominant vascular anomaly. In KTS, lymphatic malformations are predominant and may manifest as lymphatic blisters and lateral veins underlies the skin. MRI may reveal lymphatic anomalies within muscle tissue, and genetic testing often identifies mutations in PIK3CA (Fig. 2.A-C) [6]. In DCMO, the predominant feature is CM itself, presenting as erythema without increased skin temperature. MRI findings are confined to the dermis and subcutaneous tissues, with no muscle involvement, and GNA11 mutations are typically detected (Fig. 2.D-E) [7, 9]. PKWS is defined by mAVFs, often presenting with secondary venous malformations and CMs, usually accompanied by increased skin temperature and RASA1 mutations [10]. In the present case, the patient exhibited syndromic CM-LLD, and elevated skin temperature in the vascular region, alongside a medical history of progressive hyperpigmentation, pain, and ulceration—symptoms consistent with steal blood phenomena and rich arterial blood due to mAVFs. Genetic analysis confirmed a loss-of-function mutation in RASA1(p.Arg398*). These clinical, radiological, and genetic findings supported a diagnosis of Parkes-Weber syndrome.

**Fig. 3:**
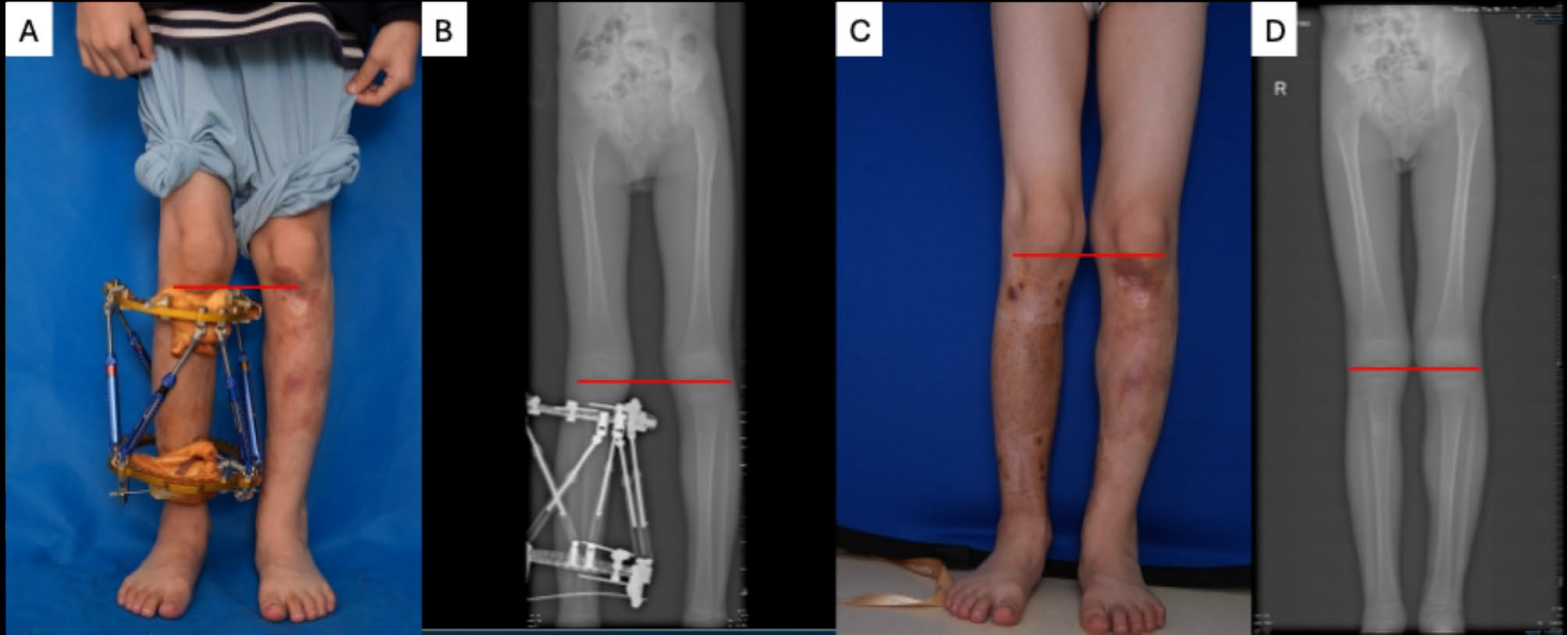
Clinical manifestation and radiograph of the patient outcome. **A:** The leg length discrepancy was treated by Illizarov Technique where the same knee level. **B:** Digital radiography of the lower extremities showed same length tibias in lesional and healthy one. **C:** The same knee level during the patent’s post-operation clinical follow up; **D:** Same knee level in Digital radiography during the patent’s post-operation clinical follow up

Management of PKWS is complex and requires a multidisciplinary approach involving interventional radiology, targeted chemotherapy, and orthopedic procedures. The pathophysiology involves aberrant vascular signaling and increased blood flow, which can stimulate bone and soft tissue hypertrophy. Genetic mutations affecting both vascular and skeletal development are implicated. The therapeutic goal is twofold: to control the primary vascular defect (mAVFs) and to alleviate the secondary condition (LLD). In this case, the patient underwent multiple interventional embolization via arterial route by using absolute ethanol and trametinib, a MEK inhibitor of the Ras/Raf/MAPK pathway. Following these treatments, the skin temperature of the vascular lesion decreased, and the LLD remained stable at 3.5 cm over two years. To prevent progression of scoliosis, limb-lengthening was subsequently performed on the contralateral(healthy) leg using the Ilizarov technique. The patient was monitored closely for 6months, and both LLD and scoliosis resolved after 3-month treatment. No recurrence of LLD or scoliosis was observed during ossification. After removal of the Ilizarov equipment, the LLD remained corrected at the 6-month follow-up (Fig. 3.A-D).

Clinically, the coexistence of CM-LLD should raise suspicion for a syndromic capillary malformation. Early multidisciplinary evaluation is essential not only to address cosmetic concerns but also to mitigate musculoskeletal complications and guide individualized treatment plans, ranging from orthotic support to surgical correction. The clinical significance of the CM–LLD association lies in the necessity of early diagnosis and multidisciplinary management. Patients with concurrent CM-LLD require comprehensive assessment—including radiologic study and genetic sequencing—to determine the extent of vascular and skeletal involvement.

Treatment strategies may include interventional embolization, targeted chemotherapy, orthopedic interventions such as limb lengthening, and long-term monitoring to address functional impairments and prevent secondary complications, such as scoliosis or osteoarthritis.

Understanding the association between CM-LLD is essential to ensure timely intervention, optimize functional outcomes, and improve quality of life for individuals with Parkes-Weber syndrome.

## Conclusion

Parkes-Weber Syndrome is a disorder characterized by the main defect of micro-arteriovenous malformation with leg length discrepancy. Parkes-Weber syndromes should be considered when patients present with leg length discrepancy and erythema of higher skin temperature in the lower extremities. Differentiate the diagnosis of Parkes-Weber syndromes by the main defect: micro-arteriovenous malformation. Therapy of multi-disciplinary team should be provided to treat Parkes-weber Syndrome to intervene the vascular malformation as well as the progressive scoliosis.

## Data Availability

No datasets were generated or analysed during the current study.

## References

[1] Kalish JM, Biesecker LG, Brioude F, Deardorff MA, Di Cesare-Merlone A, Druley T, Ferrero GB, Lapunzina P, Larizza L, Maas S, Macchiaiolo M, Maher ER, Maitz S, Martinez-Agosto JA, Mussa A, Robinson P, Russo S, Selicorni A, Hennekam RC. Nomenclature and definition in asymmetric regional body overgrowth. Am J Med Genet A. 2017;173(7):1735–8. 28475229 10.1002/ajmg.a.38266 PMC5932268

[2] Jacobs AH, Walton RG. The incidence of birthmarks in the neonate. Pediatrics. 1976;58(2):218–22. 951136

[3] *ISSVA Classification for Vascular Anomalies*. 2025.

[4] Duffy KA, Davidson RS, Kalish JM. Understanding Syndromic Leg Length Discrepancy J Pediatr. 2021;234:16–8. 33571479 10.1016/j.jpeds.2021.01.076

[5] Lee JW, Chung HY, Cerrati EW, Waner M. The natural history of soft tissue hypertrophy, bony hypertrophy, and nodule formation in patients with untreated head and neck capillary malformations. Dermatol Surg. 2015;41(11):1241–5. 26506066 10.1097/DSS.0000000000000525

[6] Maari C, Frieden IJ. Klippel-Trénaunay syndrome: the importance of geographic stains in identifying lymphatic disease and risk of complications. J Am Acad Dermatol. 2004;51(3):391–8. 15337982 10.1016/j.jaad.2003.12.017

[7] Lee MS, Liang MG, Mulliken JB. Diffuse capillary malformation with overgrowth: a clinical subtype of vascular anomalies with hypertrophy. J Am Acad Dermatol. 2013;69(4):589–94. 23906555 10.1016/j.jaad.2013.05.030

[8] Stefanov-Kiuri S, Fernandez-Heredero A. Images in clinical medicine. Parkes Weber syndrome. N Engl J Med. 2014;371(22):2114. 25427114 10.1056/NEJMicm1312948

[9] Couto JA, Ayturk UM, Konczyk DJ, Goss JA, Huang AY, Hann S, Reeve JL, Liang MG, Bischoff J, Warman ML, Greene AK. A somatic GNA11 mutation is associated with extremity capillary malformation and overgrowth. Angiogenesis. 2017;20(3):303–6. 28120216 10.1007/s10456-016-9538-1 PMC5511772

[10] Revencu N, Boon LM, Mulliken JB, Enjolras O, Cordisco MR, Burrows PE, Clapuyt P, Hammer F, Dubois J, Baselga E, Brancati F, Carder R, Quintal JM, Dallapiccola B, Fischer G, Frieden IJ, Garzon M, Harper J, Johnson-Patel J, Labreze C, Martorell L, Paltiel HJ, Pohl A, Prendiville J, Quere I, Siegel DH, Valente EM, Van Hagen A, Van Hest L, Vaux KK, Vicente A, Weibel L, Chitayat D, Vikkula M. *Parkes Weber syndrome, vein of Galen aneurysmal malformation, and other fast-flow vascular anomalies are caused by RASA1 mutations.* Hum Mutat, 2008. 29(7): pp. 959– 65. 10.1002/humu.20746 18446851

